# The Use of Deep Eutectic Solvents for the Synthesis of Iron Oxides Nanoparticles: A Driving Force for Materials Properties

**DOI:** 10.1002/chem.202500089

**Published:** 2025-03-31

**Authors:** Francesco Gabriele, Roberta Colaiezzi, Andrea Lazzarini, Franco D'Orazio, Valeria Daniele, Giuliana Taglieri, Nicoletta Spreti, Marcello Crucianelli

**Affiliations:** ^1^ Department of Physical and Chemical Sciences University of L'Aquila L'Aquila Italy; ^2^ Consorzio Interuniversitario Nazionale per la Scienza e Tecnologia dei Materiali (INSTM) Firenze Italy; ^3^ Department of Industrial Engineering, Information and Economy University of L'Aquila, Piazzale Ernesto Pontieri, Monteluco di Roio L'Aquila Italy

**Keywords:** Deep Eutectic Solvents, Hematite, IONs, Magnetite, Structural oriented synthesis

## Abstract

In this study, we explored the use of Deep Eutectic Solvents (DESs) as a green and sustainable alternative for the synthesis of Iron Oxide Nanoparticles (IONs). Six different binary mixtures of Hydrogen Bond Acceptors (HBAs) and Donors (HBDs) were prepared and thoroughly characterized to investigate how their components and physicochemical properties influence the structure, morphology, and magnetic properties of the resulting IONs. In addition, the role of DESs was assessed using ATR‐MIR spectroscopy, providing insights into HBA‐HBD interactions with iron precursors. The study highlights the critical role of DES constituents, particularly the interactions between HBAs and HBDs, in directing nanoparticle size, structure, and morphology. Indeed, our results demonstrate that the choice of DES significantly impacts the crystalline phase of iron oxide nanoparticles, yielding either magnetite (Fe₃O₄) or hematite (α‐Fe₂O₃). These findings established a robust framework for leveraging DES in nanomaterial synthesis, paving the way for more environmentally friendly approaches in diverse industrial and scientific applications.

## Graphical abstract

Deep Eutectic Solvents (DESs) structurally directed the growth of technologically relevant Iron Oxide Nanoparticles (IONs) towards magnetic (magnetite) or non‐magnetic (hematite) crystal structures, according to their intrinsic properties.

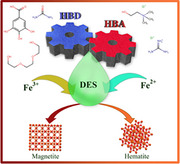

## 1 Introduction

Magnetic Iron Oxide Nanoparticles (IONs) have been extensively studied in the past years, leading to a vast number of studies.^[^ ^1^ ^]^ The fields of application explored for such systems are incredibly wide, including Magnetic Resonance Imaging (MRI),^[^ ^2^ ^]^ theranostic,^[^ ^3^ ^]^ anti‐bacterial applications,^[^ ^4^ ^]^ environmental remediation,^[^ ^5^ ^]^ agricultural,^[^ ^6^ ^]^ and catalysis employ.^[^ ^7^ ^]^ Even though magnetic nanoparticles draw most of the attention, their non‐magnetic counterpart is also at the basis of a large number of relevant uses.^[^ ^8^ ^]^ Due to the numerous applications, different types of synthetic approaches have been attempted throughout the years, leading to different material properties according to the final use.^[^ ^9^ ^]^ Among them, the most employed ones are chemical‐based methods, varying from the hydro‐/solvothermal ones,^[^ ^10^ ^]^ to thermal decomposition,^[^ ^1^, ^11^ ^]^ sol‐gel,^[^ ^1^ ^]^ and microemulsion methods.^[^ ^12^ ^]^ Considering the first methodology (which is also the most employed), there is a constant need to improve the economic and environmental performances of the solvents. Independently from the solvent employed, any kind of metal nanoparticle synthesis needs a basic environment. Being water the greenest, cheapest, and most available solvent for IONs preparation, its use presents the non‐negligible problem of wastewater treatment necessary to avoid the dispersion of alkali in the environment, thus decreasing the overall technical and economic feasibility of the process. Thus, the use of green, yet non‐water‐based solvents, might solve both problems. Deep Eutectic Solvents (DESs) represent a growing class of green solvents that could potentially fit in solving such issues. They are a relatively novel class of solvents that have gained considerable interest in recent decades due to their ease of preparation, safety, and customizable properties. Since 2003, when Abbott first introduced this class of solvents,^[^ ^13^ ^]^ studies on the properties and applications of DESs as green solvents, have significantly increased.^[^ ^14^ ^]^ DESs are typically mixtures of two or more compounds, generally Hydrogen Bond Acceptors (HBAs) and Donors (HBDs) in a certain ratio, that allows them to form a homogeneous liquid phase with a melting point lower than that of the individual components (i.e: eutectic). However, according to a more recent definition, to be classified as a DES, a mixture of compounds must deviate from ideal behavior, in the solid‐liquid phase diagram, in terms of melting temperature, composition, or both.^[^ ^14^, ^15^ ^]^ DESs found application in many fields of chemistry such as synthesis and biocatalysis,^[^ ^16^ ^]^ CO_2_ capture,^[^ ^17^ ^]^ restoration of cultural heritages,^[^ ^18^ ^]^ drug delivery,^[^ ^19^ ^]^ or separation and extraction processes.^[^ ^20^ ^]^

DESs have also been demonstrated to be an excellent green alternative to traditional solvents used for the solvothermal synthesis of nanostructured materials. Indeed, they have been successfully employed for the synthesis of nanoparticles.^[^ ^21^ ^]^ However, their use for such applications is still limited and mostly related to choline chloride:urea Type III DES employed as a classic solvent,^[^ ^22^ ^]^ or to other types of metal salts‐based DESs that have to go through decomposition to form the desired nanoparticles.^[^ ^23^ ^]^ The synthesis of IONs using DESs as solvents is present in the literature, either when dealing with non‐magnetic materials (i.e., α‐Fe_2_O_3_),^[^ ^24^ ^]^ or with magnetic/superparamagnetic ones.^[^ ^25^ ^]^ Despite the huge potentialities of DES, their use in synthetic procedures is still underexploited, considering either their use as a pure solvent,^[^ ^21^, ^25^ ^]^ or directly as a metal precursor to generate nanoparticles through DES decomposition.^[^ ^24^, ^25^ ^]^ Even in these cases, the overall solvent properties, which might influence and directly drive the synthetic outcomes toward the desired material, are not yet fully analyzed. Furthermore, the use of chloride:urea Type III DES often spreads its shadow over the discovery and the use of new DESs.

In this work, we wanted to tackle and untangle the potential effect that DES can have in chemical and material synthesis. Thus, we prepared and characterized a series of six DESs (some of them never reported in the literature), obtained by varying the functional groups of the HBA–HBD couple. Afterward, we successfully employed the obtained green solvents to dissolve iron salts, aiming to the formation of oxide nanoparticles through precipitation by pH variation, expecting the formation of superparamagnetic nanoparticles. However, once noted that not all the prepared materials were responding to an external magnetic field, we provided a thorough physicochemical and magnetic characterization of the nanoparticles, with the goal of relating their properties with the ones of the solvents. To the best of our knowledge, very few attempts to correlate DES properties with synthetic outcomes of the reactions in which are employed are present in the specialized literature, so far. With this work, we tried to set up a robust methodology to unveil the role of DES in material preparation, thus paving the way for a wider employ of such green solvents.

## 2 Experimental

### 2.1 Chemicals

FeCl_2_⋅4H_2_O (98%) and FeCl_3_⋅6H_2_O (97%) were provided by Alfa Aesar; KOH (98%) was purchased from Carlo Erba; EtOH, *p*‐nitroanisole, *p*‐nitroaniline, Nile red, choline chloride (ChCl), triethylene glycol (TEG), urea (U), gallic acid (GA), and guanidine chloride (GC) were purchased from Merck; deionized water was obtained by reverse osmosis by means of a Decasei Forwater apparatus.

### 2.2 Preparation of DES

Six different eutectic mixtures were prepared by mixing ChCl or GC as HBAs with GA, TEG, or U as HBDs. All mixtures were heated to 90°C under magnetic stirring overnight to evaluate which binary mixture formed a homogeneous liquid. In particular, ChCl:U and ChCl:TEG were prepared at mole ratios of 1:2 and 1:3, respectively, as reported in the literature.^[^ ^13^, ^26^ ^]^ Instead, the eutectic diagram of the other four DESs was determined by varying the mole fraction of their constituents by means of Differential Scanning Calorimetry (DSC) using a Mettler Toledo DSC 3 (Mettler Toledo, Columbus, Ohio, USA) differential scanning calorimeter equipped with an Intracooler METTLER TOLEDO TC100 MT. Indium (melting point at 156°C) and water (melting point at 0°C) were used as standards to calibrate the DSC calorimeter for both cooling and heating scans. About 10 mg of samples were placed in pierced‐lid aluminum pans. The pure components were heated at 5°C/min until their fusion transition was complete, according to each melting transition reported by the provider. Regarding the mixtures of HBA and HBD, they were first kept at −90°C for 20 min and then heated at 5°C/min up to 100°C under a nitrogen atmosphere with a flow rate of 50 mL/min. The analysis was performed on ChCl:GA, GC:TEG, and GC:U, respectively, by analyzing at least three different molar fractions of each binary mixture to determine their eutectic ratio.^[^ ^13^, ^27^ ^]^

### 2.3 Karl Fischer Titration

The water content of all DESs was measured using a HI933 Karl Fischer titrator from Hanna Instruments, Cluj Napoca, Cluj (Romania), with the two‐component reagents Hydranal Titrant 5 and Hydranal NEXTGEN Solvent FI from Honeywell. First, the water content of the methanol was determined, after which 1.5 g of methanol was added to 1.5 g of DESs. The solutions were then vigorously stirred to achieve a homogeneous mixture before injecting approximately 1 g of each solution into the titrator chamber.

### 2.4 Physicochemical Properties of DESs

The density of each eutectic solvent was determined by pouring the DES into 5 mL volumetric flasks, which were held in a thermostatic bath for 30 min. The samples were then adjusted to volume by removing excess DES with a Pasteur pipette. After cooling the flasks to room temperature, they were weighed on an analytical balance. The ionic conductivity of the eutectic mixtures was measured using an Analytical Control–ORION Research conductivity meter with a platinum cell (cell constant *K* = 1.05 cm^−1^). Viscosity measurements were carried out using a Fungilab Viscolead mod. ADV L rotational viscometer equipped with a temperature sensor.

All physicochemical properties were recorded in duplicate over a temperature range from 50°C to 90°C.

### 2.5 Measure of the Solvatochromic Parameters of DESs

The Kamlet–Taft parameters (*α*, *β*, and π*) and polarity (*E*_NR_) could provide valuable insight into the role of DESs in guiding the synthesis of iron oxide nanoparticles. In fact, α and β describe the ability of the DESs as Lewis's acid or base, while π* represents their polarizability.^[^ ^28^ ^]^ These parameters were determined for all DESs using three different solvatochromic probes by using the Equations ((1), (2), (3))^[^ ^29^ ^]^

(1) $$\pi^{*}=14.57-\frac{4270}{\lambda_{\max,\mathrm{OMe}}}$$

(2) $$\alpha =\frac{\left(19.9657-1.024\times \pi^{*}-\frac{10^{4}}{\lambda_{\max,\mathrm{NR}}}\right)}{1.6078}$$

(3) $$\beta =\left(11.134-\frac{3580}{\lambda_{\max,NH_{2}}}-1.125\times \pi^{*}\right)$$

where *λ*_max__,__OMe_, *λ*_max,NR_, and *λ*_max,NH2_, are the maximum wavelength of absorption of, in the order, *p*‐nitroanisole, Nile red, and *p*‐nitroaniline.

Furthermore, the Nile Red was also used to determine their polarity by means of the Equation (4)

(4) $$E_{\mathrm{NR}}=\frac{28591}{\lambda_{\max,\mathrm{NR}}}$$

The solvatochromic probes were dissolved in methanol at appropriate concentrations: 10 mM for Nile Red and 200 mM for both *p*‐nitroanisole and *p*‐nitroaniline. Then, 25 µL of each probe solution was poured into a glass vial and kept at 60°C. After the methanol had completely evaporated, approximately 2 g of DES was added to each vial and stirred until the probe was fully dissolved. UV/VIS spectra of the DES‐probe mixtures were acquired in duplicate using a Shimadzu UV‐160 A instrument equipped with a Peltier thermostat set at 60°C to avoid the crystallization of the DESs.

### 2.6 Synthesis of IONs

Once the various DESs were prepared, we proceeded with the synthesis of IONs by adapting a literature procedure.^[^ ^30^ ^]^ Briefly: 0.275 g of FeCl_3_⋅6H_2_O (1.02 mmol) and 0.150 g of FeCl_2_⋅4H_2_O (0.75 mmol) were added to a sealed vial containing 2 g of previously prepared DES and kept under stirring at 80°C for 20 min. Afterward, 0.335 g of solid KOH (5.97 mmol) was added to the reaction mixture and left to react for 90 min. Then, the reaction solution was transferred inside a falcon and centrifuged for 30 min at 5000 rpm to separate the formed particles from the solvent. Once the supernatant was removed, we proceeded to wash the oxide nanoparticles three times with ammonia solution (pH ≈ 9) and three times with EtOH, centrifuging at 5000 rpm for 30 min after each wash. The obtained nanoparticles were then dried overnight at 80°C inside an air oven and then calcined in air at 550°C for 3 h (*T*_ramp_ = 2.5°C/min) in a muffle furnace.

The synthetic procedure described above was repeated for each DES that was prepared and characterized.

### 2.7 Attenuated Total Reflectance‐Mid Infrared Spectroscopy (ATR‐MIR)

ATR‐MIR measurements were performed by means of a PerkinElmer Spectrum Two instrument equipped with an atmospheric UATR Two accessory and a DTGS detector. Each spectrum was the result of an eight scan average, collected with 4 cm^−1^ resolution in the range of 4000–500 cm^−1^.

### 2.8 Powder X‐Ray Diffraction (PXRD)

The crystalline structure of the samples was investigated by means of Powder X‐Ray Diffraction (PXRD) technique, using a PW3050/60 X'Pert PRO MPD diffractometer from PANalytical equipped with Cu K_α_ (wavelength *λ* = 0.154 nm) radiation source in Debye–Scherrer geometry. Patterns were collected with a sample stage spinner, putting ca. 200 mg of powder into a zero‐background sample holder. Data are shown in a 2θ range from 20° to 70°, where the main peaks of iron oxide structures are present. Crystal average dimension was estimated by applying the Scherrer Equation.^[^ ^31^ ^]^

### 2.9 Scanning Transmission Electron Microscopy (STEM)

Morphological studies were carried out by Scanning Transmission Electron Microscopy (STEM), using a FESEM ZEISS Gemini500 instrument. Images were acquired either with transmitted or backscattered electrons, and operating with an accelerating voltage of 20 kV. The samples were dispersed via sonication in methanol; afterward, a drop of the suspension was deposited on a TEM copper grid (200 mesh) with an amorphous carbon film, and measured after full evaporation of the dispersion solvent.

### 2.10 Alternating Gradient Magnetometry (AGM)

Magnetization curves were obtained by means of a PMC Micromag 2900 alternating gradient magnetometer from Lakeshore Cryotronics. The samples were weighted and placed inside a homemade sample holder. The measurements were performed at room temperature by varying the magnetic field in the range of −12 kOe to +12 kOe.

## 3 Results and Discussion

### 3.1 DES Characterization

According to the most recent definition of DESs, this subclass of the eutectics has to deviate from the ideal behavior; that can be estimated by Equation 5

(5) $$\ln\left(x_{i}y_{i}\right)=\frac{\Delta H_{m}}{R}\times \left(\frac{1}{T_{m}}-\frac{1}{T}\right)$$

where *R* is the universal gas constant, Δ*H_m_*, and *T_m_* represent the enthalpy and temperature of melting of the pure compounds while *x_i_* and *γ_i_* denote the molar fraction and the activity coefficient of the component *i*, respectively.^[^ ^15^, ^32^ ^]^ The value of the activity coefficient can be approximated to 1 by assuming the ideal behavior of the liquid mixture.^[^ ^33^ ^]^

Due to the decomposition of choline chloride, which begins concurrently with its melting point, it is not possible to estimate the ideal behavior of its eutectic mixtures except by indirectly determining its fusion enthalpy.^[^ ^32^ ^]^ Similarly, the endothermic peak of GA centered at 275°C was associated with its decomposition rather than its melting.^[^ ^34^ ^]^

Therefore, in this work, the eutectic diagrams of the DESs studied were determined and overlapped, when it was possible, with their ideal behavior as shown in Figure S1. The only exceptions regard the well‐known mixtures choline chloride:urea (ChCl:U)^[^ ^13^ ^]^ and choline chloride:triethylene glycol (ChCl:TEG),^[^ ^27^ ^]^ whose eutectic points were already clearly determined.

Regardless of the molar fraction investigated, no liquid phases were observed for the mixture composed of GC and GA (GC:GA) up to a temperature of 363 K, making it not suitable for the selected iron nanoparticles synthesis conditions (*T*_react_ = 353 K). In contrast, the combination of choline chloride and GA (ChCl:GA) resulted in a clear liquid. For this mixture, the eutectic composition is 3:1, with a melting temperature of 315 K (Figure S1a) similar to that obtained by Picchio et al.^[^ ^35^ ^]^ However, as aforementioned, due to the decomposition of the pure components, it was not possible to classify this mixture as classic eutectic or DES. Although some works have already reported the application of this DES, to the best of our knowledge, none has determined its eutectic diagram.^[^ ^35^, ^36^ ^]^

Figure S1b shows the ideal behavior of the GC:TEG indicating that the ideal eutectic mixture is mainly composed of TEG (*χ*_TEG_ = 0.93) with only a 2 K depression in the melting temperature compared to pure TEG. Regarding the experimental phase diagram, it was possible to determine the branch related to the excess of GC up to the eutectic point. This is probably due to the thermal properties of the glycol‐based DESs, which often show an amorphous behavior with the disappearance of some characteristic thermal transitions.^[^ ^27^, ^37^ ^]^ However, despite a high molar fraction of TEG, only the thermal transition ascribable to the eutectic was observed at around 200 K, while the eutectic composition appears to be at a molar ratio of about 1:2. This result not only highlights the significant decrease in the melting temperature compared to the ideal behavior, but also a shift in the molar fraction of the eutectic composition.

Lastly, the DES formed between GC and U (GC:U) shows a molar ratio of 1:2, which slightly differs from the ideal mixture (Figure S1c) and matches the one of the DES ChCl:U.^[^ ^13^ ^]^ In addition, a significant decrease in melting temperature from 361 K (ideal mixture) to 327 K (real mixture), is observed. Similar results have been obtained by Parnica and Antalik who first proposed this mixture in 2014 as DES with a melting temperature of 331 K at the same molar ratio identified in this work.^[^ ^38^ ^]^

Physicochemical properties of DESs vary significantly with their water content, which cannot be directly estimated from that of the starting materials, as the eutectic solvents were prepared by maintaining the binary mixture at 90°C overnight. Therefore, determining the effective water content of each DES is essential, especially when hydrophilic constituents are used. The results of the Karl Fischer analysis showed that all the DESs studied were characterized by a low water content, which ranges around 1%.

**Figure 1:**
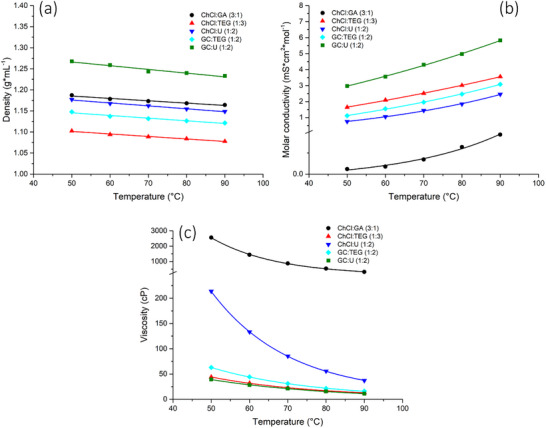
Behavior of density (a), molar conductivity (b), and viscosity (c), of the five studied DESs between 50°C and 90°C: ChCl:GA (3:1) (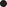), ChCl:TEG (1:3) (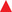), ChCl:U (1:2) (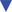), GC:TEG (1:2) (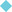), and GC:U (1:2) (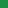).

Figure 1 shows the behavior of the densities, conductivities, and viscosities of the five DESs investigated between 50°C and 90°C. Since all the studied DESs consist of a salt as the HBA and a neutral HBD, which differ significantly in both molar ratio and molecular weight, the molar conductivity was reported as a function of temperature. The molecular weight of each DES was calculated by summing the molecular weights of the pure constituents, each multiplied by its molar coefficient, as reported in Equation 6 ^[^ ^27^, ^39^ ^]^

(6) $$MW_{DES}=n_{\mathrm{HBA}}\;MW_{\mathrm{HBA}}+n_{HBD}MW_{\mathrm{HBD}}$$

where *n*_HBA_ and *n*_HBD_ represent the molar coefficients of the HBAs and donors, respectively.

The density of all studied DESs slightly decreases as the temperature increases (Figure 1a), due to thermal expansion of the solvent volume. The observed linear decrease in density appears to affect all DESs similarly, with their trends being nearly parallel to each other across the investigated temperature range.

Both molar conductivity and viscosity of DESs are indices of the mass transfer ability of the solvent. As evident from the Figure 1b, the molar conductivity of the DESs increases with increasing temperature due to an increment in the thermal agitation.

Regardless of temperature, ChCl:GA results to be the least conductive DES, with molar conductivity values that never exceed 0.1 mS⋅cm^2^⋅mol^−1^. On the contrary, GC:U is the most conductive DES among the five investigated, with values that increase with temperature from 3 to 6 mS⋅cm^2^⋅mol^−1^. The other three DESs appear to have similar molar conductivity values, ranging between 0.75 and 3.5 mS⋅cm^2^⋅mol^−1^. In particular, the trends observed for ChCl:U and ChCl:TEG in this study are consistent with those reported in other works.^[^ ^13^, ^27^ ^]^

As shown in Figure 1c, the viscosity of DESs decreases with increasing temperature, exhibiting an exponential trend opposite to that observed for molar conductivity. As expected, the ChCl:GA mixture is the most viscous among the five DESs, with values comprised between 2600 cP and 300 cP ranging from 50°C to 90°C. Despite ChCl:U results to be the second most viscous DES, its viscosity is consistently ten times lower than that of ChCl:GA, across the investigated temperature range. Several studies deal with the characterization of reline (ChCl:U (1:2)), highlighting how the water content can strongly affect its viscosity. Indeed, a wide range of viscosities, from approximately 120 cP to 220 cP at 50°C, is reported by comparing the results presented in different papers.^[^ ^13^, ^40^ ^]^ Also the behavior of the ChCl:TEG mixture is in complete accordance with the results obtained in our previous work, showing a decrease from 44 cP at 50°C down to 13 cP at 90°C.^[^ ^27^ ^]^

Lastly, both guanidine‐based DESs result in very low viscous solvents among the five investigated. The viscosity of GC:TEG varies from 63 to 16 cP between 50°C and 90°C, slightly higher than that of its choline derivate, ChCl:TEG, probably due to the lower content of TEG in the guanidine‐based solvent. On the other hand, GC:U DES was almost 10 times less viscous compared to ChCl:U, despite sharing the same mole fraction between HBA and HBD. This difference could be related to the lower dimension and greater mobility of the guanidine cation compared to the choline cation.

**Figure 2:**
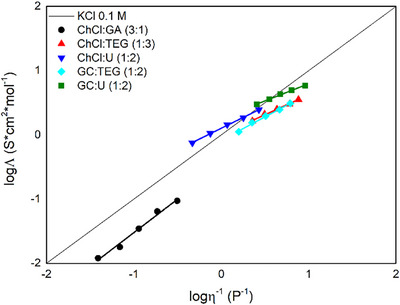
Walden plot of ChCl:GA (3:1) (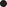), ChCl:TEG (1:3) (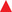), ChCl:U (1:2) (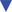), GC:TEG (1:3) (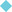), and GC:U (1:2)ChCl:GA (3:1) (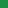); the black straight line is referred to a 0.1 M solution of KCl.

By plotting the logarithm of the molar conductivity against the logarithm of the fluidity (the reciprocal of viscosity) the Walden plot can be obtained. This graph qualitatively represents the ionicity of DESs, allowing us to compare these solvents depending on the anion–cation interaction of the HBA salt component. Figure 2 presents the Walden plot of the five DESs studied in this work, with an ideal behavior of a diluted KCl solution (0.1 M) included as a threshold between poor and good ionic solvents.

As evident in the Walden plot, the ChCl:GA mixture lies in the poor ionic region indicating that the charges of the anion and cation are strongly bound. In contrast, all other mixtures more or less align with the behavior of KCl aqueous solution. The two choline‐based DESs (ChCl:TEG and ChCl:U), exhibit similar behaviors to those reported in the literature, with the former being a poor ionic solvent and the latter a slightly better ionic solvent.^[^ ^27^, ^39^ ^]^

Interestingly very similar ionicity were observed by substituting the choline ion with the guanidine ion. Despite its higher fluidity, the GC:U DES behaves similarly to ChCl:U falling within the regions defined by the KCl behavior depending on the temperature. The similarity between the two DESs composed of TEG is even more pronounced, as their plots almost completely overlap. This finding suggests that very similar interactions are established between TEG or U and the chlorine ions, which appear to be independent of the cation.

### 3.2 Kamlet–Taft Parameters and Polarity of the DESs

To fully characterize the DESs proposed in this work, we investigated how they interact with a solute by determining the Kamlet–Taft parameters and the polarity as well. Table 1 summarizes the values of the empirical parameters π*, *α*, *β*, and *E*_NR_ calculated by means of the Equations ((1), (2), (3), (4)) reported in the Experimental Section.

**Table 1:** Mean values of the Kamlet–Taft parameters π*, *α*, *β*, and polarity (*E*_NR_), assessed spectrophotometrically using solvatochromic probes measured at 60°C, and calculated by means of Equations ((1), (2), (3), (4)).

DES	π*	*α*	*β*	*E*_NR_
ChCl:GA (3:1)	1.18 ± 0.06	1.10 ± 0.06	1.01 ± 0.02	48.5 ± 0.1
ChCl:TEG (1:3)	1.12 ± 0.03	0.47 ± 0.02	0.74 ± 0.03	51.7 ± 0.2
ChCl:U (1:2)	1.21 ± 0.03	0.94 ± 0.01	0.50 ± 0.03	49.3 ± 0.1
GC:TEG (1:2	1.21 ± 0.03	0.55 ± 0.03	0.50 ± 0.02	51.0 ± 0.1
GC:U (1:2)	1.25 ± 0.03	0.92 ± 0.03	0.50 ± 0.03	49.2 ± 0.2

The results presented in Table 1 show that all DESs are quite similar in terms of polarizability and polarity, which range from 1.12 to 1.25 and from 48.5 to 51.7, respectively. However, the values of *α* and *β* vary significantly depending on the constituents of the DESs. Indeed, the higher value of *α* and *β* of the ChCl:GA mixture is probably ascribable to the presence of the three hydroxyl groups and the carboxylic moiety of the GA, along with the hydroxyl group of the choline, which together allow the formation of an extensive hydrogen bond network.

The two TEG‐based DESs show a noticeable difference in their ability to accept hydrogen bonds, as indicated by the *β* parameter. This difference may be due to the lower TEG content in the GC:TEG mixture compared to ChCl:TEG (1:2 and 1:3, respectively). However, the low content of TEG does not affect the *α* value, probably because the hydroxyl groups of the glycol are directly involved in the supramolecular structure of the DESs, contributing less to establish other hydrogen bonds as donors.

The Kamlet–Taft parameters of the ChCl:U DES are in complete accordance with those reported in the literature,^[^ ^41^ ^]^ and are very similar to those of GC:U DES. Being all nitrogen atoms of the guanidine cation involved in resonance, it is logical to hypothesize that their ability to act as HBAs is limited, which would explain the low *β* value.

### 3.3 IONs Syntesis and Characterization

Once DESs were prepared and characterized, we synthesized the IONs reported in Table 2, aiming to achieve a magnetic phase similar to that obtained in our previous works via hydro‐ and solvothermal syntheses.^[^ ^2^, ^7^ ^]^

**Table 2:** List of the Iron Oxide Nanoparticles (IONs) prepared by using different Deep Eutectic Solvents (DES) as reaction media.

Entry	Sample name	DES
1	ION‐1	ChCl:U (1:2)
2	ION‐2	ChCl:TEG (1:3)
3	ION‐3	ChCl:GA (3:1)
4	ION‐4	GC:U (1:2)
5	ION‐5	GC:TEG (1:2)
6^[^ ^a^ ^]^	–	GC:GA

Abbreviations: ChCl = choline chloride; GA = gallic acid; GC = guanidine chloride; TEG = triethylene glycol; U = urea.

^[a]^ The sample corresponding to Entry 6 could not be prepared, since the ratio for the eutectic mixture of GC:GA DES was not found under any of our working conditions.

**Figure 3:**
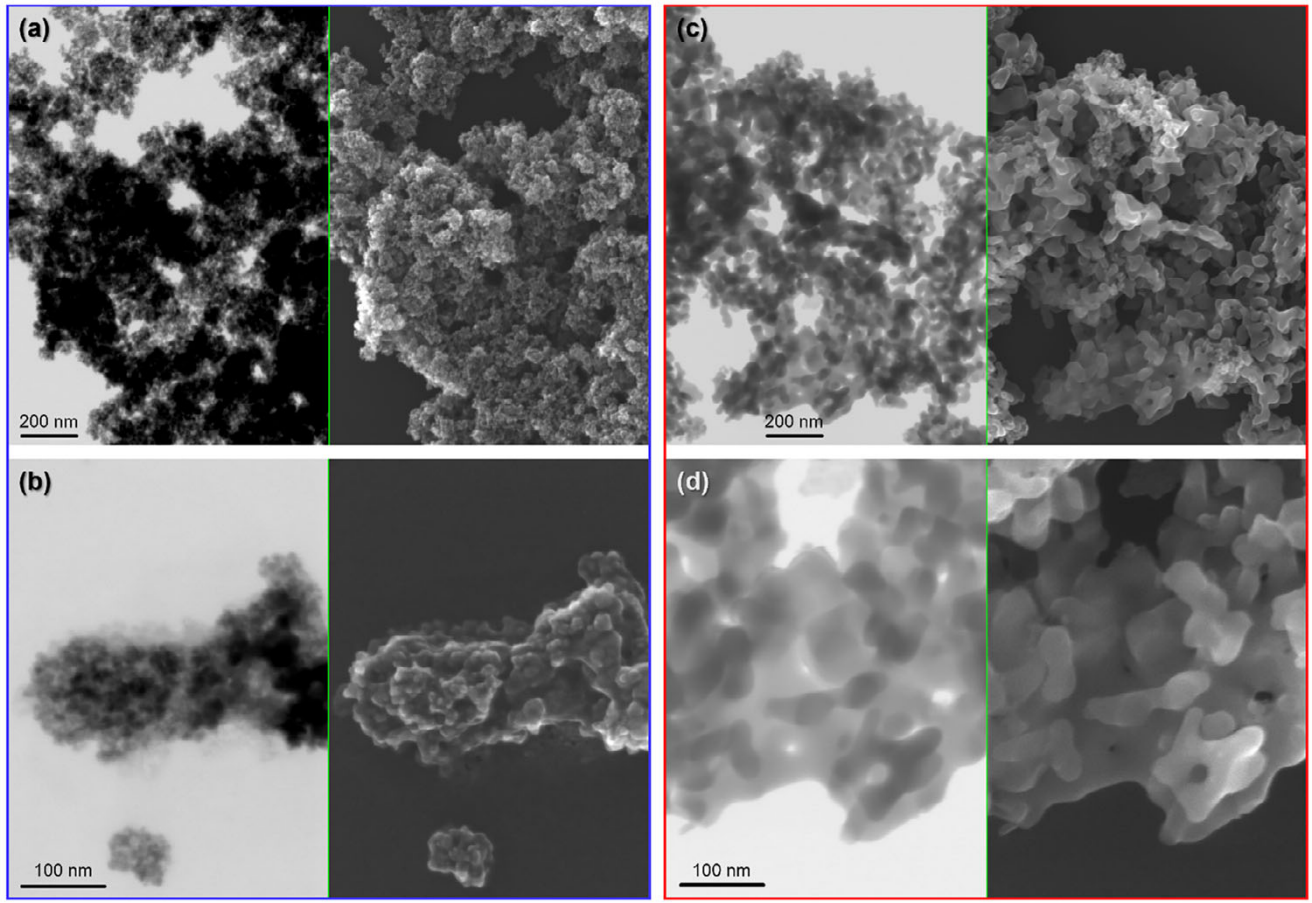
SEM images collected simultaneously in transmission (left side of each insert) and with backscattered electrons (right side of each insert) of samples ION‐1 (highlighted in blue) and ION‐2 (highlighted in red). In Parts a–c samples are displayed with a 50 kx magnification; in Parts b–d the same samples are displayed with a 150 kx magnification.

After the synthetic step, we first observed the morphology of the samples by means of electron microscopy. We immediately observed a trend, namely that, depending on the solvent employed for the synthesis, we ended up with two well‐distinguished families of particles. Indeed, for the sample ION‐1 reported in Figure 3a–b (as well as for ION‐4 and ION‐5, reported in Figures S3 and S4, respectively) we observed small, regular, round‐shaped nanoparticles, similar to those obtained in our previous research.^[^ ^2^, ^7^ ^]^ Conversely, for sample ION‐2 reported in Figure 3c–d (as well as for ION‐3, reported in Figure S2), the particles appear to be much larger, elongated, and interconnected.

After such observation, we wanted to understand if the differences in morphology would also reflect on the crystal structure of the materials. Therefore, we proceeded to analyze the synthesized IONs with PXRD.

**Figure 4:**
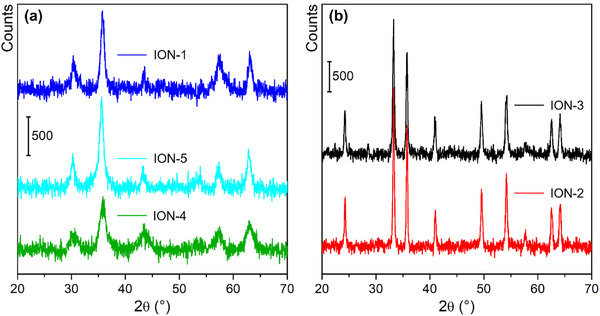
PXRD patterns of IONs samples reported according to their crystal structure: magnetite (part a) and hematite (part b).

Diffraction data reflect perfectly the differences evidenced by electron microscopy. Indeed, samples with smaller round particles possess the cubic spinel crystal structure of magnetite (ICSD 82237 – Figure 4a),^[^ ^42^ ^]^ while the materials with larger and elongated particles have the hexagonal structure of hematite (ICSD 130951 – Figure 4b).^[^ ^43^ ^]^ By simply observing the average peak broadening, it is evident that the two families of samples differ not only in structure but also in crystal size. However, we completed PXRD analysis by calculating the average crystal size of IONs by using the Scherrer Equation,^[^ ^31^ ^]^ finding that samples with magnetite structure have quite lower particle size values, in the range of 5 to 10 nm; IONs samples with hematite structure, instead, have a larger average crystal dimension, around 25 nm (all average particles dimension data are reported in Table 3). Diffraction data presented above need some more insights to assign the structural differences observed to the solvents employed, as the prepared IONs are obtained through different synthetic steps, onto which we need to focus singularly. The first one is the addition of KOH to the Fe ionic solution: the addition of a strong base to iron salts is known to form Fe(OH)_2_ and Fe(OH)_3_, depending on the oxidation state of the precursors.^[^ ^44^ ^]^ However, when in contact at T > r.t., they directly combine in solution, leading to the formation of magnetite (Fe_3_O_4_).^[^ ^44^ ^]^ In the case only Fe(III) hydroxide is formed, upon heating to high T (above 500°C), it will dehydrate progressively leaving hematite (α‐Fe_2_O_3_) as the resulting iron compound.^[^ ^45^ ^]^ Thus, the two presented hypotheses perfectly match with the obtained results. To further strengthen these findings, the reported Fe‐O phase diagrams indicate that once α‐Fe_2_O_3_ and Fe_3_O_4_ are formed, they are thermally stable and non‐miscible up to 1500°C, making the phase transformation of one into the other unrealistic.^[^ ^46^ ^]^ Therefore, the only reason standing behind the structural differences in the prepared IONs might stand in the different solvents employed.

**Table 3:** Summary of the structural and magnetic properties of the synthesized IONs.

Sample name	Structure type	Particles average dimension (nm)^[^ ^a^ ^]^	Saturation magnetization (emu∙g^−1^)	Magnetic coercivity (Oe)
ION‐1	Magnetite (Fe_3_O_4_)	8.5 ± 0.2	48.0	−10.2
ION‐2	Hematite (α‐Fe_2_O_3_)	26.7 ± 0.6	1.9	−79.8
ION‐3	Hematite (α‐Fe_2_O_3_)	22.1 ± 0.6	0.8	−397.6
ION‐4	Magnetite (Fe_3_O_4_)	5.1 ± 0.1	45.6	−0.6
ION‐5	Magnetite (Fe_3_O_4_)	10.8 ± 0.3	49.3	−10.3

^[a]^ Calculated with Scherrer equation D=Kλ/βcosθ; with *K* (shape factor) = 0.9, *λ* (Cu Kα) = 0.15406 nm, *β* = FWHM of the most intense diffraction peak (in radians), *θ* = diffraction angle of the most intense peak.

Even if from the point of view of chemical bonding we are dealing with two iron oxides, also ATR‐MIR (see Figure S5) helped to detect small differences between hematite and magnetite. The main difference that we encountered, lays in the vibrational frequency of the Fe─O bond. In fact, we observed a shift in frequency from the 519 cm^−1^ of the hematite samples (i.e., ION‐2 and ION‐3, red and black curves in part b of Figure S5, respectively),^[^ ^47^ ^]^ to the 560 cm^−1^ of the magnetite ones.^[^ ^48^ ^]^

**Figure 5:**
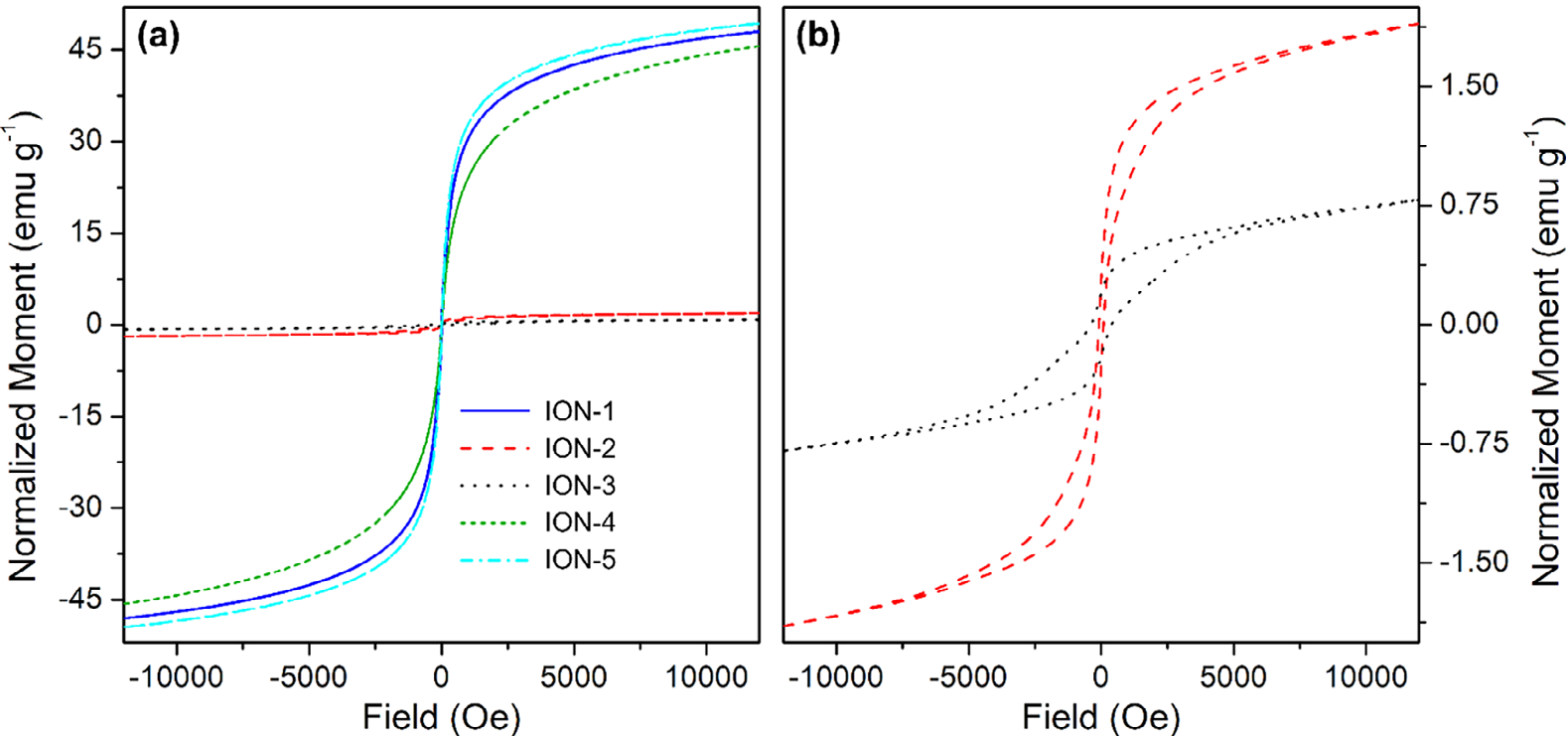
Part (a): AGM curves of the whole series of IONs samples. Part (b): magnification of the magnetization curves relative to samples ION‐2 (red dash) and ION‐3 (black dots).

The ultimate difference between magnetite and hematite samples is their magnetic response. The samples with magnetite structure and small crystal size display the typical superparamagnetic behavior expected for such materials (see Figure 5a, blue, green, and cyan curves), with a magnetization that has not yet reached its maximum at 12 kOe, and the complete absence of magnetic hysteresis.^[^ ^49^ ^]^ Furthermore, slight differences in crystal size are evidenced in the maximum magnetization value reached by each sample, in agreement with the slightly different shapes of the magnetization curves. In particular, larger particles show higher initial susceptibility (the slope of the curve at small fields) and consequently higher maximum specific magnetic moment reached at 12 kOe, consistent with a simple Langevin model for superparamagnetism. As a result, the initial susceptibility is approximately inversely proportional to the diffraction peak full width half maximum (FWHM), as evidenced in Table 3.

Conversely, samples ION‐2 and ION‐3 having hematite structure show, as expected, a rather poor magnetic response (see Figure 5a, red and black curves respectively).^[^ ^50^ ^]^ Some residual magnetic components can still be observed in these two samples, as shown in the magnification of Figure 5b, suggesting the presence of a small amount of magnetic iron oxide phase. Furthermore, the existence of magnetic hysteresis is due to the larger particle size and their increased interconnection.

After the thorough physicochemical and structural characterization of the prepared ION samples, it is evident that the DES employed for the preparation of the materials is not innocent towards the synthetic outcomes. However, the measurements of density, viscosity, and conductivity, used to build the Walden plot for each solvent are not sufficient to evidence a clear relationship between the used DES and IONs properties. Therefore, further investigation into their nature has been made, in order to explain their effect in directing the structural growth of our IONs.

### 3.4 DES Influence on IONs Synthesis Precursors

**Figure 6:**
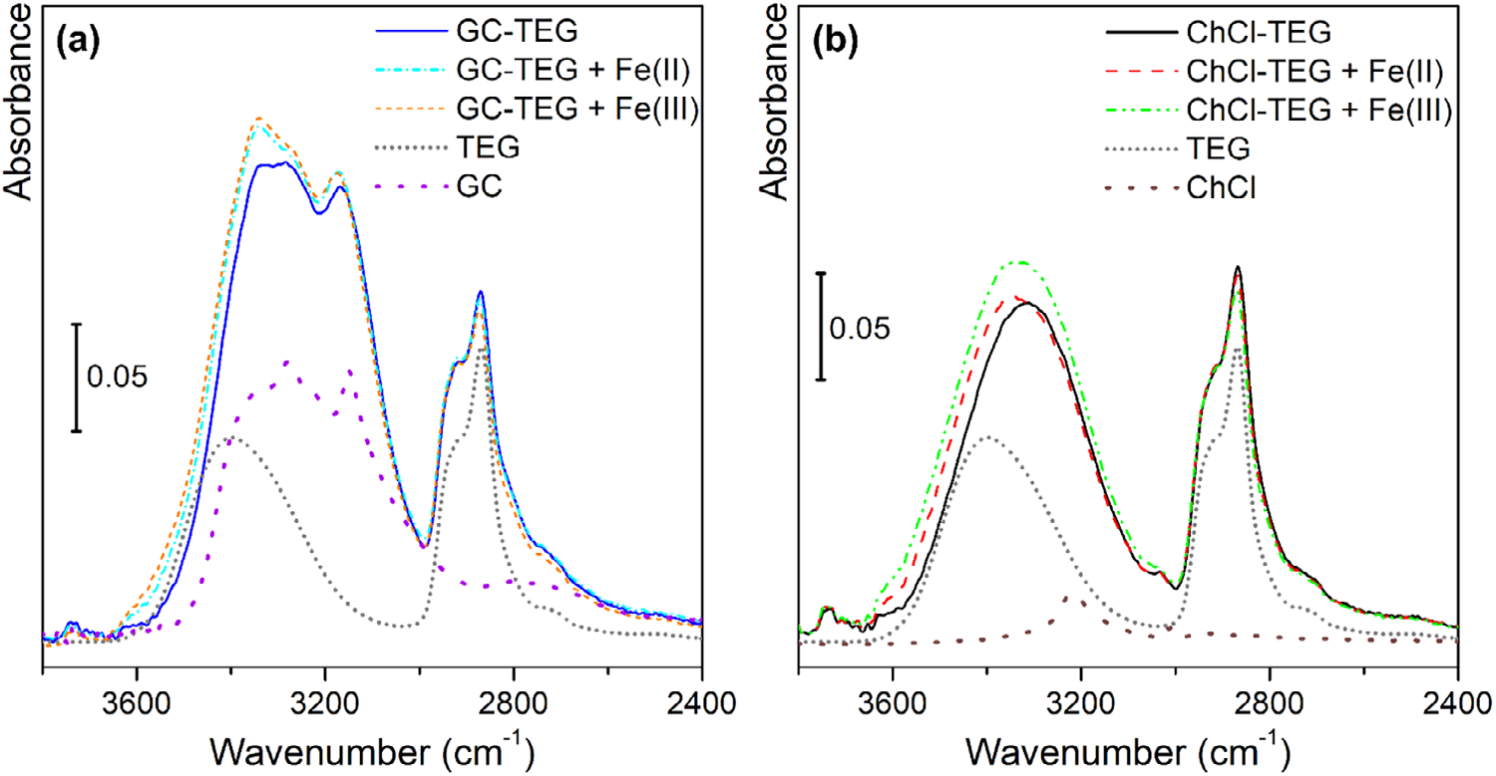
Part (a): ATR‐MIR spectra (normalized with respect to the 1060 cm^−1^ peak of TEG) of GC:TEG (1:2) alone (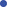), in interaction with FeCl_2_∙4H_2_O (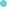), and in interaction with FeCl_3_∙6H_2_O (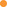). Part (b): ATR‐MIR spectra (normalized with respect to the 1060 cm^−1^ peak of TEG) of ChCl:TEG (1:3) alone (∙), in interaction with FeCl_2_∙4H_2_O (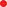), and in interaction with FeCl_3_∙6H_2_O (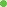). Dotted curves are referred to DES components, namely TEG (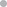), GC (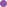), and ChCl (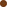). Full spectra are reported in Figure S6.

The structure of crystalline materials (such as iron oxides) is mainly directed during the synthetic process. To better understand the chemical interactions taking place between the solvents and the nanoparticle precursors, we performed ATR‐MIR measurements of the pure DES and after the addition of the iron salts used for the synthesis. In particular, we focused on two DESs, namely GC:TEG (1:2) and ChCl:TEG (1:3). This choice was made since the change in HBA directed the syntheses towards different structures (with GC we obtained Fe_3_O_4_, while with ChCl we obtained α‐Fe_2_O_3_). Spectroscopically, even if most of the spectrum of the DESs remains unaffected (see Figure S6), in the ν(OH) region (displayed in Figure 6a–b), we observed meaningful changes.

Considering GC:TEG (1:2) solvent (blue curve in Figure 6a), it is interesting to notice how the functional groups of its constituents behave. In particular, it is evident how in the DES the vibrations related to ν_symm_(NH_2_) and ν_antisymm_(NH_2_) of GA (at 3280 and 3160 cm^−1^, respectively)^[^ ^51^ ^]^ are almost untouched. Contrarywise, the ν(OH) signal arising from the glycol redshifts from 3400 to 3335 cm^−1^, indicating a strong interaction consequent to the formation of the eutectic phase.^[^ ^26^ ^]^ Upon addition of hydrated iron salts (light blue and orange curves in Figure 6a), the only observed variation is a slight growth of the ν(OH) signal, due to the presence of additional water molecules; however, no significant shift in frequencies is present. When occurring, the blue shift of this signal (moving towards the frequency of free TEG) would mean a weakening of the interaction between HBA and HBD.^[^ ^26^ ^]^ Indeed, GC:TEG DES behaves as a traditional solvent, leaving the direction of the structural evolution exclusively to the base added to allow nanoparticle precipitation, leading to magnetite as the final result.

Instead, the blue‐shifting behavior of ν(OH) signal is well visible for ChCl:TEG (1:3) solvent (black curve in Figure 6b): in fact, upon DES formation we observe a lowering of its wavenumber value, going from 3400 to 3310 cm^−1^. After the addition of iron precursors (red and green curves in Figure 6b), the position of the same vibration moves back towards higher wavenumbers, namely at 3340 cm^−1^, indicating a loosening of the HBA‐HBD interaction. In this way, ChCl is more free to interact with Fe salts, thus directing the synthesis towards the formation of hematite, as already documented in the literature.^[^ ^24b^ ^]^

## 4 Conclusions

In this paper, we successfully demonstrated that DESs can be a valid alternative to traditional solvents when employed in the solvothermal synthesis of nanoparticles. Their nature makes them intrinsically active in directing the structural growth of the materials. Indeed, the choice of the proper HBA:HBD couple proved to be crucial for controlling the crystalline phase. We focused on the synthesis of IONs: either their magnetic (magnetite, Fe₃O₄) or non‐magnetic (hematite, α‐Fe₂O₃) crystal structure is the basis for a plethora of diverse applications. Such structural directional growth is often achieved by using templating agents/molecules that must be removed post‐synthetically, which is completely avoidable in the preparation process when employing DES. Switching to a green solvent that is not water‐based also avoids the post‐synthetic treatment of mother liquors, necessary to neutralize pH and removing leftover metal ions before water is released back into the environment.

The characterization of the DES highlighted that not all solvents behave equally in directing the structural growth of the nanoparticles. For instance, the H‐bond acceptance capability (Kamlet–Taft parameter *β* > 0.5) along with a weaker HBA:HBD interaction strength, observed spectroscopically in DES interacting with iron precursors, showed to be responsible for directing the structure of growing nanoparticles towards hematite. Despite this observation correlates quite well with experimental data, a full explanation of this phenomenon is not possible to be given yet, and requires further investigations.

These findings not only underscore the potential of DES as a green and sustainable solvent but also pave the way for further studies on optimizing their properties for specific applications in the synthesis of nanostructured material. This may be of relevant interest when dealing with the synthesis of mixed‐metal magnetic nanoparticles, which nowadays rely on diverse procedures depending on the selected species to be alloyed with iron ones,^[^ ^52^ ^]^ potentially simplifying the overall procedure. Not only, the recent discovery and development of DESs directly possessing a basic component might also avoid the addition of an external base, making the handling of the processes at the industrial level potentially safer and easier. The methodological approach developed in this work will provide a significant contribution to a deeper understanding of the role of DES in chemical synthesis, promoting their wider use both in industrial and scientific fields.

## Conflict of Interests

The authors declare no conflict of interest.

## Supporting information



Supporting Information

## Data Availability

The data that support the findings of this study are available from the corresponding author upon reasonable request.
