# Supplementary material for: The Use of Deep Eutectic Solvents for the Synthesis of Iron Oxides Nanoparticles: A Driving Force for Materials Properties
Source: Chemistry. 2025 Mar 31;31(25):e202500089. doi: 10.1002/chem.202500089 (PMC12057613; doi:10.1002/chem.202500089)
Supplement: Supplementary file 1 — Supporting Information [file CHEM-31-e202500089-s001.pdf]

# SUPPORTING INFORMATION

# The Use of Deep Eutectic Solvents for the Synthesis of Iron Oxides Nanoparticles: a Driving Force for Materials Properties

Francesco Gabriele[a,†], Roberta Colaiezzi[a,†], Andrea Lazzarini[a,b,*], Franco D'Orazio[a], Valeria Daniele[c], Giuliana Taglieri[c], Nicoletta Spreti[a], Marcello Crucianelli[a,b,*]

[a] Department of Physical and Chemical Sciences, University of L'Aquila, Via Vetoio ("A.C. De Meis" and "R. Ricamo" buildings), 67100 L'Aquila, Italy

[b] Consorzio Interuniversitario Nazionale per la Scienza e Tecnologia dei Materiali (INSTM), Via Giuseppe Giusti 9, 50121 Firenze, Italy

[c] Department of Industrial Engineering, Information and Economy, University of L'Aquila, Piazzale Ernesto Pontieri, Monteluco di Roio, 67100 L'Aquila, Italy

[†] The authors contributed equally to this research paper.

[*] Corresponding authors: andrea.lazzarini@univaq.it; marcello.crucianelli@univaq.it

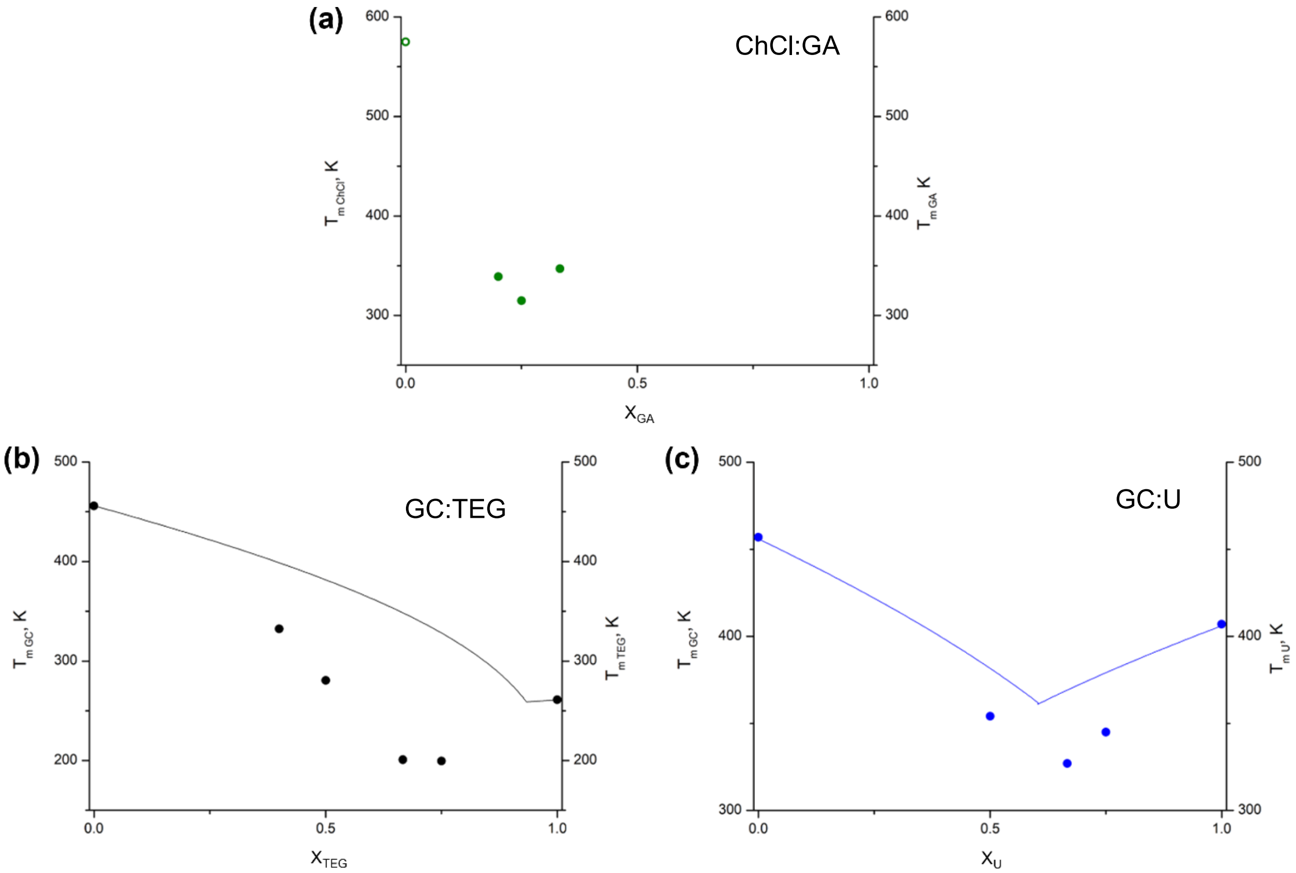


**Figure S1.** Phase diagram of the DESs ChCl:GA (a), GC:TEG (b), and GC:U (c) in which the solid lines represent their ideal behavior (calculated by equation 5). The open circle in the (a) graph indicates the onset temperature of choline chloride in which the melting of the salt and its decomposition starts.

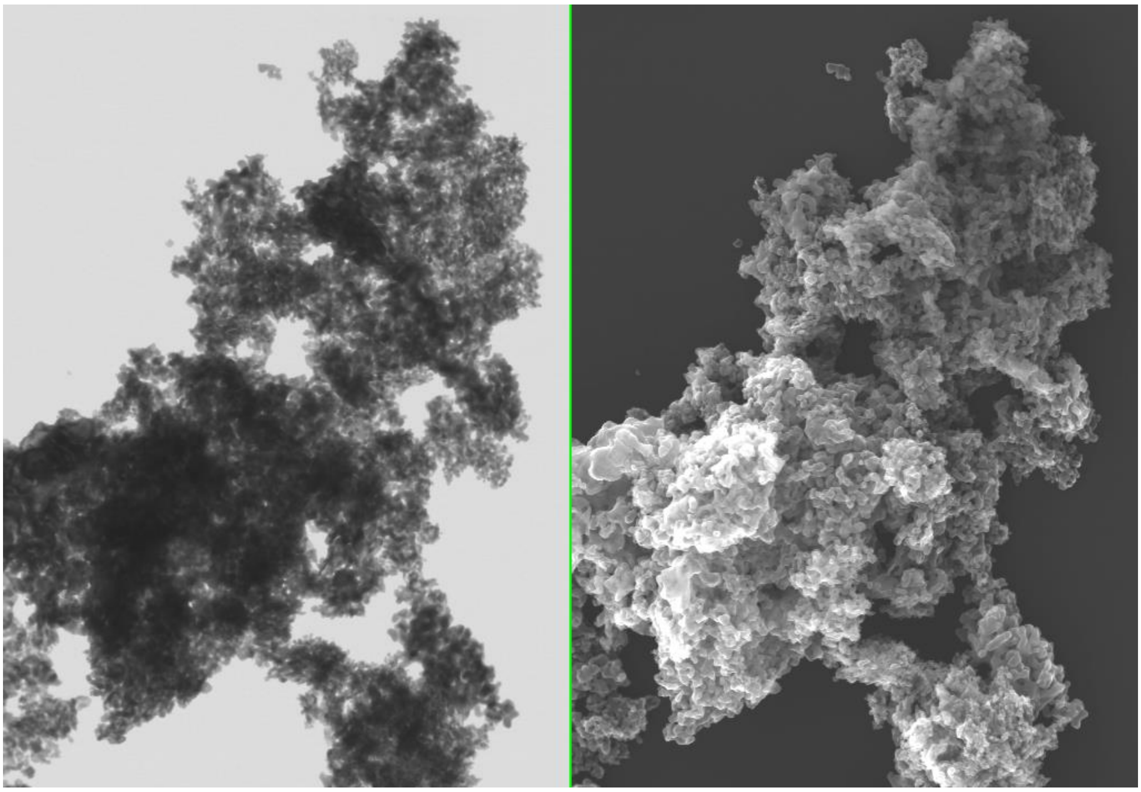

**Figure S2.** SEM image (20 kx magnification) collected simultaneously in transmission (left) and with backscattered electrons (right) of samples ION-3.

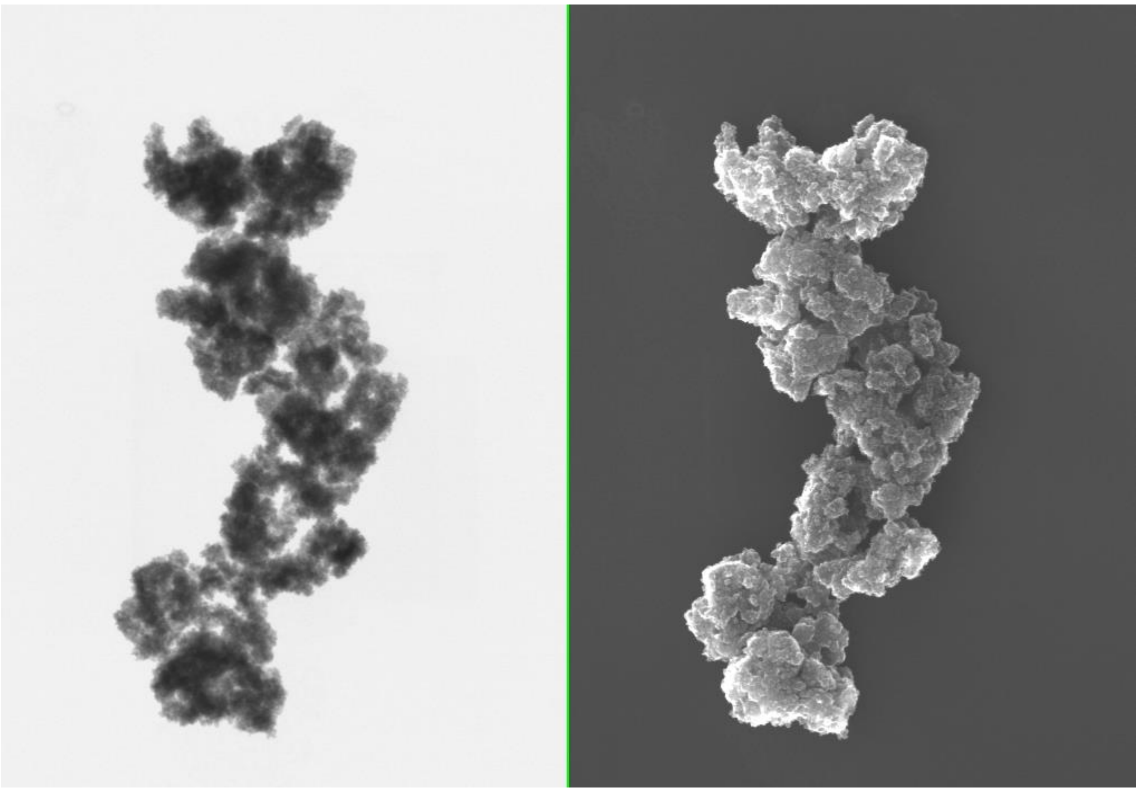

**Figure S3.** SEM image (20 kx magnification) collected simultaneously transmission (left) and with backscattered electrons (right) of samples ION-4.

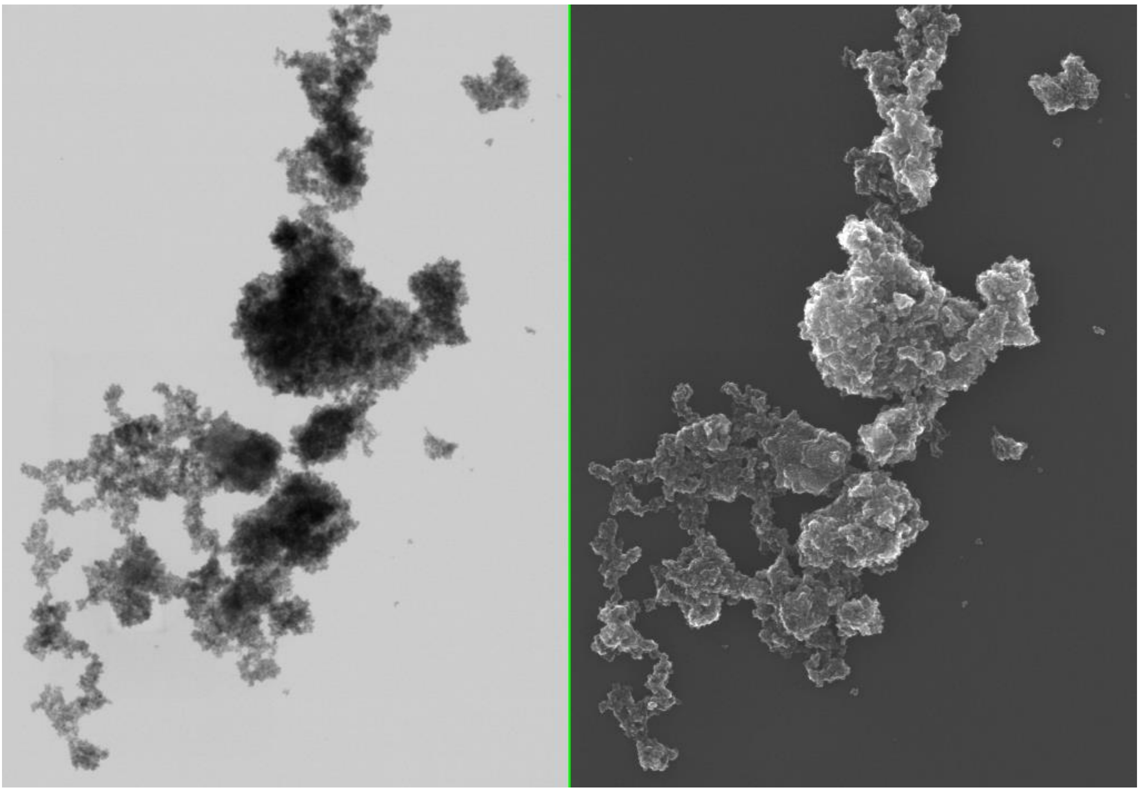

**Figure S4.** SEM image (20 kx magnification) collected simultaneously in transmission (left) and with backscattered electrons (right) of samples ION-5.

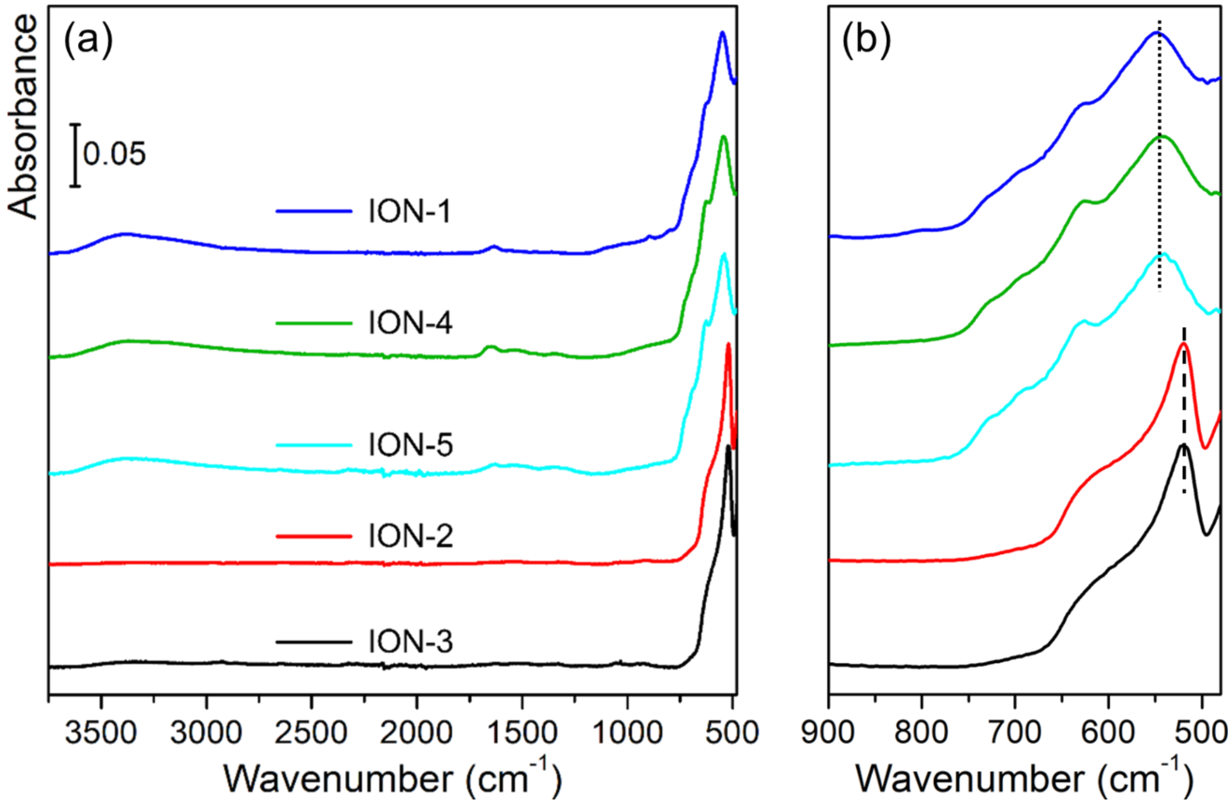


**Figure S5.** Part (a): ATR-MIR full spectra of the whole series of IONs samples. Part (b): spectral magnification in the Fe−O stretching region; dotted line highlights the signal at 560 $cm^{-1}$, dashed line highlights the signal at 519 $cm^{-1}$.

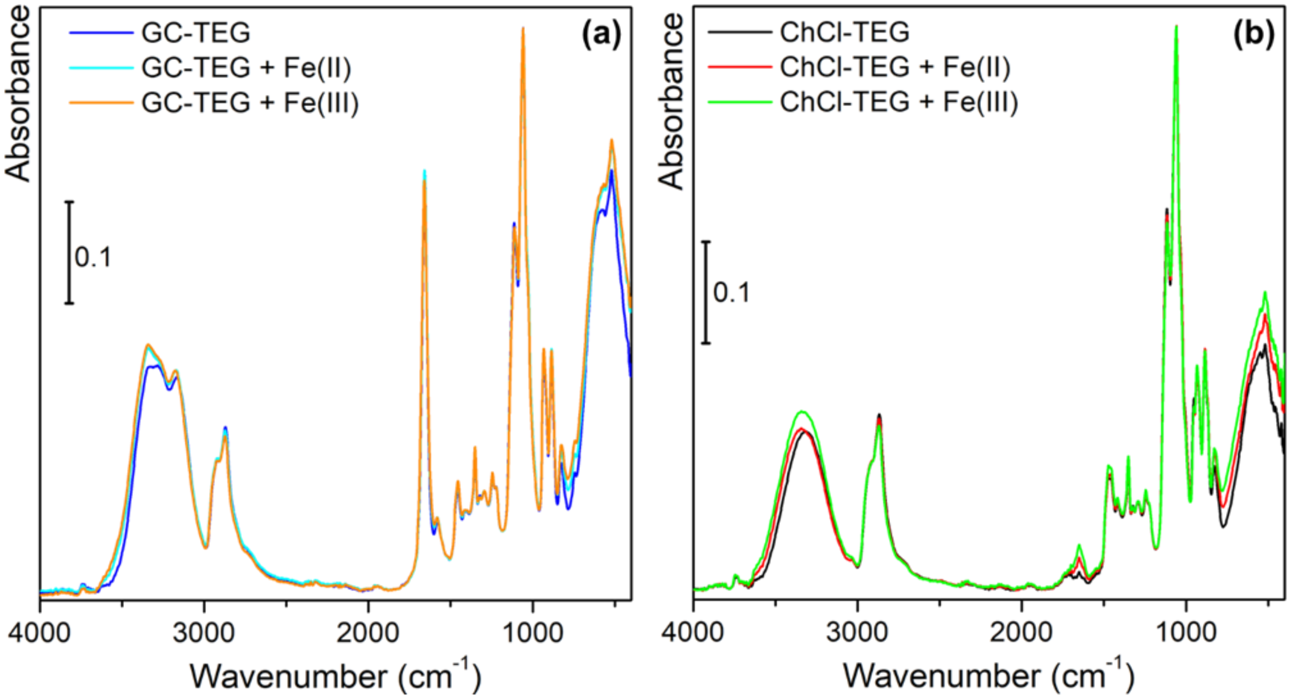


**Figure S6.** ATR-MIR full spectra (normalized with respect to the 1060 $cm^{-1}$ peak of TEG) of different DES, namely GC:TEG (1:2) in part (a), and ChCl:TEG (1:3) in part (b), in interaction with the Fe(II) and Fe(III) salts used for ION samples syntheses.
